# The Development of a Surface Finisher of Car Park Slab Using Waterborne Silicon Acrylic with Polyamide [Part I: Performance Evaluation]

**DOI:** 10.3390/ma12010171

**Published:** 2019-01-07

**Authors:** Hoseong Jeong, Junho Gong, Wanseok Yoon, Dooyong Cho

**Affiliations:** 1 Department of Convergence System Engineering, Chungnam National University, Daejeon 34134, Korea; hsjeong@cnu.ac.kr (H.J.); jhgong@cnu.ac.kr (J.G.); 2 Highway and Transportation Technology Institute, Korea Expressway Corporation, GyeongGi-Do 18489, Korea; yws@ex.co.kr

**Keywords:** surface finisher, car park, waterborne silicon acrylic, polyamide, performance evaluation, eco-friendly

## Abstract

A waterborne coating system for car park slab has recently gained interest as an alternative for solvent-based finishing materials due to environmental concerns and prolongation of service life. However, water-based finishers, regardless of their eco-friendly properties, have relatively lower hardness compared to traditional finishing systems. In order to overcome this obstacle, a hybrid technology was used to develop a substitute surface finisher for car park slab and its performance was evaluated according to the KS (Korean Standard) F 4937. Initially, the proper mix ratio of polyamide was found by comparing adhesion via pull-off-test results and other performance evaluation tests. From the test results, it was found that mixing polyamide with silicon acrylic finisher caused an increase in adhesion strength. Silicon acrylic with a 30% mix ratio of polyamide resin (SA+PR30%) was selected to perform the rest of the tests and the results satisfied the acceptance criteria of KS F 4937 and were compared with a recent water-based polyurethane finisher with cementitious powder (WPC). Finally, it was verified that the developed finisher could be an alternative finisher of urethane and epoxy finishers as it has good mechanical properties and emit less volatile organic compounds (VOC).

## 1. Introduction

While concrete pavements in underground car parks are required for high durability, they raise environmental concerns due to fine dusts generated by continuous frictional loads between automobile tires and the concrete. Additionally, the dust would not be properly filtered because of a lack of air circulation systems underground. The surface finishing materials have been widely used on the pavements in order to improve the environment of underground car parks and to provide convenience to the users [1].

**Figure 1:**
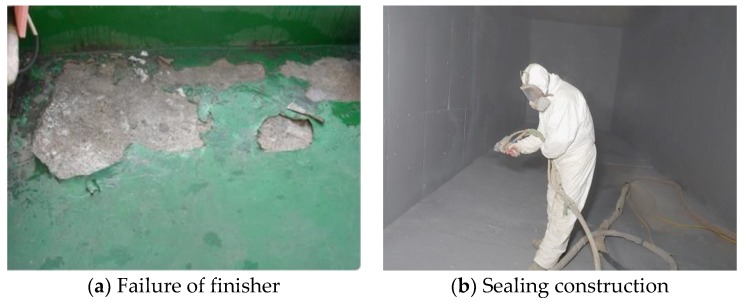
Structural and environmental issues of surface finishers.

The most popular finishers, which consist of urethane and epoxy, have been mainly used in car parks slab constructions since they are cheap, durable, waterproof, and nonslip [2,3]. However, there are some drawbacks when using epoxy and urethane surface finishers. In terms of structural behaviors, both repetitive tractional forces generated by sudden stops of cars and imposed impact loads can lead to fracture and spalling of epoxy or urethane finisher lamination. These types of failures generally occur to the slabs of car parks that are continuously loaded and inappropriately constructed as shown in Figure 1a [4]. Additionally, the urethane and epoxy finishers have another disadvantage in terms of health and safety issues. This is because volatile organic solvents are combined with the urethane or epoxy finishers to improve workability during construction. Uncomfortable odor and endocrine disruptors from the solvent-based coating and enclosed construction environment expose the workers to health and safety issues (Figure 1b). Additionally, toxic gases released from the solvent based finisher during construction have been known to cause fire incidents [1,3].

In order to solve such problems, various studies have been conducted to enhance the structural properties of surface finishers and mitigate the health and safety issues in construction sites. Environmentally friendly waterborne coatings have been considered as alternatives to minimize the emissions of volatile organic compounds (VOCs) and replace the classic solvent-based coatings [5,6]. In the early stage of the studies, the major disadvantage attributed to the waterborne coatings was relatively shorter service life compared to the solvent-borne coating materials [5,7]. However, a recent research attempt has been made on a hybrid of acrylic and urethane to lower the VOC content of the coating [8].

The objective of this study is to develop a surface finisher for the car park slabs by associating waterborne silicon acrylic, which is currently used in sports fields, with polyamide resin in order to minimize the defects to replace the existing surface finishers. For comparison, the conventional finishers such as epoxy, urethane, and water-based polyurethane with mixture of cementitious powder finishers (WPC) [9] were used as control groups for analyzing the performances of the developed finisher in accordance with Korean Standard (KS) F 4937: Korean standard for surface finishing material for car park slab.

## 2. The Quality Standard for Surface Finishing Material for Car Parks Slab

The epoxy and urethane surface finishers have been widely used in car park slab to improve both indoor and outdoor environment of buildings. Although they are typically applied to slab sealing construction, plenty of structural failures of the finishers and casualties that lead to economic losses have been found owing to an absence of a specific quality assurance guideline for the surface finishers for car parks slab. In order to improve these circumstances, KS F 4937 aims to standardize the evaluation and performance criteria of top finishers for car park slab and to ensure protection from health and safety issues.

The KS F 4937 consists of five different subcategories of evaluation that include structural and chemical properties as listed in Table 1 below. In the case of adhesion by pull-off test, the acceptance, which is more than 1.2 N/mm^2^, is designed to designate a common safety performance factor for sealing constructions of car park slab. Impact resistance test is planned to maintain the quality level of the surface finishers. This is because the coating system is influenced by a variety of imposed impact loads caused by falling objects, driving cars, and stamping foot. Permeability test is intended to qualify general safety factors. The abrasive wear of top coating materials is ordinarily created by foreign materials such as sand or debris on the surface of tires and top layer of finisher. Abrasive wear resistance to wheel moving test is intended to assess abrasive wear performance of the surface finishers. Finally, the pollutant emission test is fundamentally based on the ‘Indoor Air Quality Control Act’ in Korea and according to this act, it is mentioned that construction materials emitting pollutants shall not be used for flooring materials. To satisfy this condition, the objective of the emission test is to set a quality assurance guide.

Although there is a specific standard with diverse evaluation criteria for surface finishers for car parks slab in Korea, an integrated standard is not specified for surface finishers in European countries. It was found that each evaluation criterion from KS F 4937 is allocated in different designated standards as listed in Table 1 above. The European requirement of adhesion by pull-off test requires higher strength compared to the Korean counterpart and different requirements are demanded depending on the test methods such as the impact resistance test, the permeability test, and the abrasive wear resistance to wheel-moving test. Additionally, the pollutant emission test is excluded in the European standard.

## 3. Materials and Test Methods

### 3.1. Materials

#### 3.1.1. Waterborne Silicon Acrylic Emulsion

A surface finishing material, which was previously developed with a Chinese company, was supplied by Eco Sports Chemical Technology Company (GyeongGi-do, Korea). The finisher chemically consists of Silicon Acrylic (SA) resin, water, and polyoxyethylene pentylphenol ether. Their chemical compositions are listed in Table 2 below. The SA finisher is currently applied to outdoor sports field areas.

#### 3.1.2. Urethane Resin

A urethane surface finisher was provided by Kangnam Chemical Company (Seoul, Korea) Polyurethanes are broadly implemented in various manufactures in terms of high-resilience, rigid insulation and high-performance adhesives. The chemical composition of the urethane finisher is shown in Table 3 below.

#### 3.1.3. Epoxy Resin

The epoxy surface finisher adopted for the experiment of this study was provided by Jevisco Company (Seoul, Korea). Epoxy resins are normally reacted either with themselves or with a variety of co-reactants. Additionally, because of the sustainable properties of the epoxy resins, it is possible to use in diverse areas required for chemical resistance, extreme flexibility, high strength and hardness, good heat resistance, and high electrical resistance. Two types of epoxy resin were selected for the experiment and their chemical compositions are indicated in Table 4.

#### 3.1.4. Polyamide Resin

A polyamide resin (PR) using hybrid technology with the waterborne SA emulsion was supplied by Jevisco Company (Seoul, Korea). This material is a primer, which is a mixture of modified polyamide and hardener with ratio of 4:1. It is commonly used in architectural finishing, factories, and civil engineering. The chemical composition of this material is shown in Table 5 below. The manufactured polyamides consist of numerous carbon chains in the repeating unit. Several properties of the polyamides involve high strength, abrasion resistance, resilience, and good hydrophilicity. Due to these features, the polyamides are generally applied in the manufacture of clothing and carpets. In terms of engineering plastics, they are compound with fillers, pigments, glass fiber, and toughening agents to enhance specific properties of the polymer. Reinforced plastics for vehicles and films for food packaging, for instance, have good mechanical strength and barrier properties against oxygen and oils [10].

#### 3.1.5. Water-Based Polyurethane with Mixture of Cementitious Powder Finishers (WPC)

Recently, water-based polyurethane with a mixture of cementitious powder finisher was developed by Chung-buk National University [9]. It not only satisfied the acceptance criteria of the KS F 4937 such as adhesion, permeability of water, pollutant emission, shock, and wheel move resistance test but also was tested for compressive and flexural strength and chemical resistance. From the test results, the optimal mixing ratio of the surface finishing materials containing a mixture of cementitious powder and water-based polyurethane resin were developed, as shown in Table 6 below.

### 3.2. Specimen Preparation and Test Methods

In order to define the optimized proportion of the PR for developing a top finisher and to compare its adhesive strength, the adhesion by pull-off test from the evaluation criteria in the KS F 4937 was initially performed. This was because the low adhesion strength was the critical structural factor in creating fine dust caused by a combination of fragments and continuous loads from tires. Once the optimum finisher was defined, the remaining tests of KS F 4937 proceeded for the subject to determine whether it satisfied the other evaluation criteria of KS F 4937.

#### 3.2.1. Adhesion by Pull-Off Test

**Figure 2:**
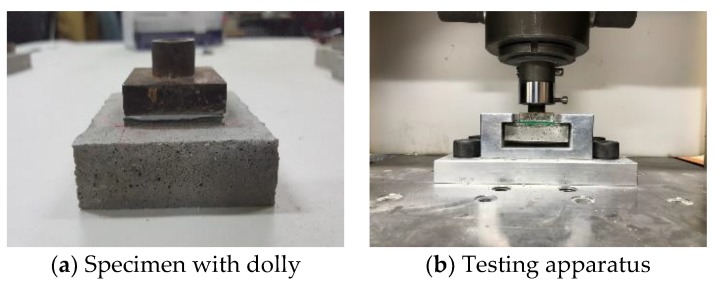
Specimen and apparatus of adhesion test.

Mortar substrates were made up with dimensions of 70 mm× 70 mm× 20 mm described in KS L ISO 679 to use for preparing specimens. The substrates were molded after 1 day and immersed in 20 °C water for 28 days. Once the substrates were completely cured, the mix design of top finishers which were epoxy and urethane, and SA with polyamide as specified in Table 7 and Table 8, respectively, were painted out 6 times with 0.5 mm thickness and dimension of 40 × 40 mm on to the surface substrates. After painting the finisher, it was cured during the two weeks in the curing room with 22 °C temperature and 45% relative humidity for 2 weeks. Dollies were attached on the top surface of the finishers as indicated in Figure 2a, and the specimens were loaded in testing apparatus as shown in Figure 2b to measure and compare adhesive strength of the surface finishers.

#### 3.2.2. Impact Resistance Test

**Figure 3:**
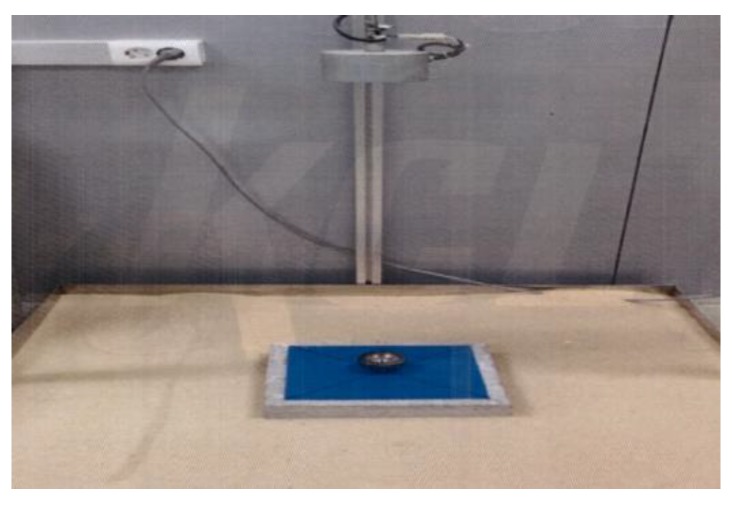
Impact resistance test apparatus.

A concrete substrate of KS F 2762: Measurement of bond strength of products for the protection and repair of concrete structure by pull-off was cast with dimensions of 300 × 300 × 50 mm as described in KS F 4937. The substrate was demolded after 1 day and immersed in 20 °C water for 28 days. The top finisher was coated on the surface of the concrete substrate and cured for 14 days. As indicated in Figure 3, the specimen was placed on leveled sand according to KS F 2221 and a steel ball (W21000 of KS B 2001) was dropped 3 times from 500 mm high. A visual observation with naked eyes was conducted after the test in order to check for pits, cracks, grounding, and spalling.

#### 3.2.3. Permeability Test

**Figure 4:**
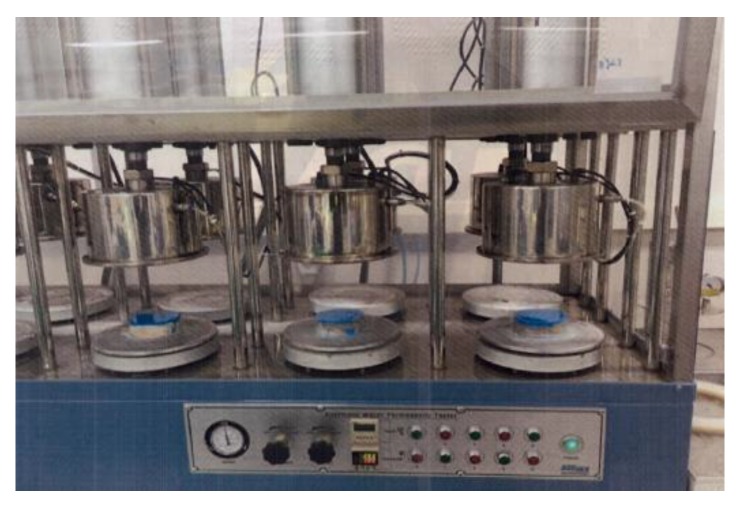
Permeability test apparatus.

The top finisher was coated on the surface of mortar substrates with a dimension of Φ100 mm × 30 mm. The specimens were placed into the permeability test apparatus, as indicated in Figure 4, and a constant water pressure head of 0.3 N/mm^2^ was applied to the sealed testing apparatus for 3 h. After finishing the test, the specimen was wiped out using filter paper for 10 s and cut in half to check water penetration through the surface of the specimens.

#### 3.2.4. Abrasive Wear Resistance to Wheel-Moving Test

**Figure 5:**
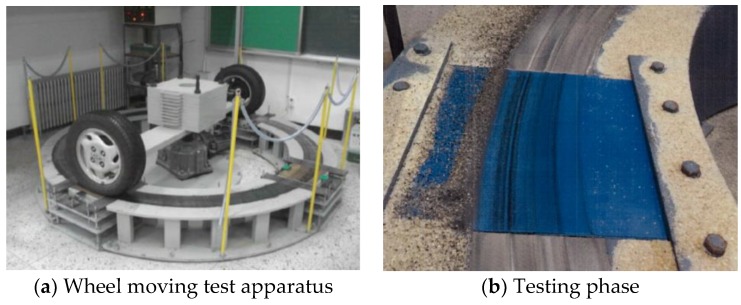
Abrasive wear resistance to wheel-moving test.

As the actual automobile tires are used for the abrasive wear resistance test, it is practical to evaluate the performance of the abrasive wear resistance of top finishers. Abrasive wear is normally caused by the materials that are in between the surface of tires and finisher and the frictional loads from tires. A concrete substrate of KS F 2762 was molded with dimensions of 300 mm × 300 mm × 50 mm and the top finisher was spread out with dimensions of 200 mm × 200 mm. As indicated in the Figure 5a, the two moving wheels were loaded with 300kg weights and cycled 80,000 times with a speed of 5 km/h. Sand was dropped every 30 cycles from 1 m high (Figure 5b), and observations were made every 10,000 cycles to check for pits, cracks, grounding and spalling. At the end of the test, 6 pieces of the samples were taken from the specimen to measure loosing surface depth caused by repetitive frictional forces from the moving tires.

#### 3.2.5. Pollutant Emission Test

**Figure 6:**
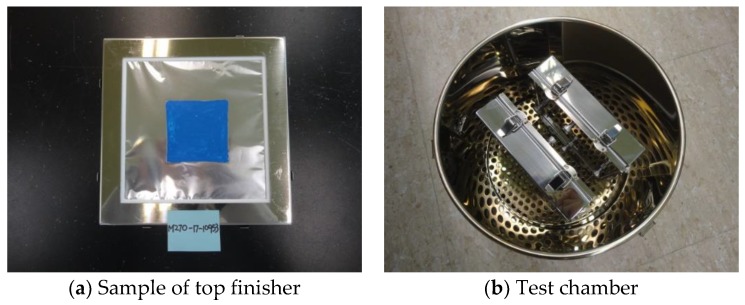
Pollutant emission test.

The top finisher was spread out on a glass plate (Figure 6a) in accordance with KS I ISO 16000-11 and dried out at 25 °C and 50% relative humidity. The test specimens were fixed using frames and placed into a small chamber for 7 days as shown in Figure 6b. Followed by determination methods which were KS I ISO 16000-3 and 16000-6, the quantities of total volatile organic compounds (TVOC), Formaldehyde and Toluene were taken for measurements.

## 4. Results

### 4.1. Adhesion by Pull-Off Test

**Figure 7:**
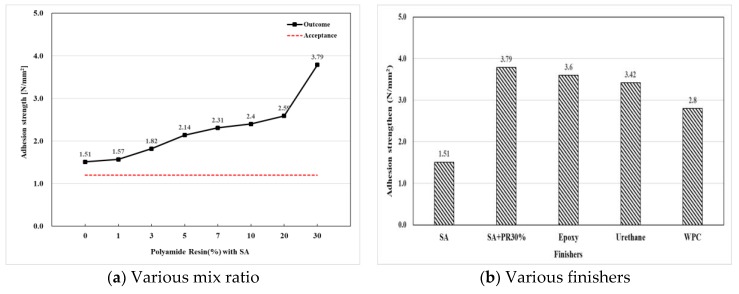
Adhesion strength.

The adhesion by pull-off test was conducted as a preliminary test in this study to determine the compatibility of a surface finisher by examining mean adhesive strength of specimens which was considered as a fundamental structural property. The results of mean adhesive strength of three specimens with different mix ratios of top finishers are indicated in Figure 7a below. All the specimens satisfied the criterion of KS F 4937 which is 1.2 N/mm^2^ and the BS (British Standard) EN 1542 which is 1.5 N/mm^2^. The outcome of the SA+PR 30% showed 2.5 times higher value compared to the silicon acrylic (SA), 1.35 times higher than the WPC, and slightly exceeded the performance of epoxy and urethane as shown in Figure 7b. Additionally, there was a directly proportional relationship between the adhesion strength and the amount of PR.

### 4.2. Impact Resistance Test

**Figure 8:**
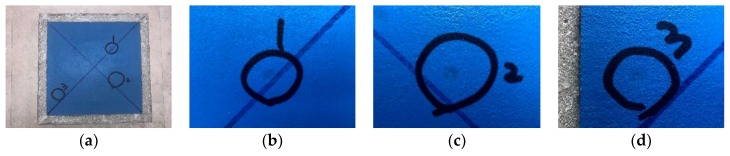
Impacting spots of steel ball.

The impact resistance test was continuously performed to determine that the developed finisher was resistant to impact load and satisfied the KS F 4937. The steel ball was dropped three times from 500 mm high above to the top finished segment of specimen as described in KS F 4937. As indicated in Figure 8b–d, there was no pit, crack, grounding, and spalling observed on the surface of the specimen. Throughout the visual inspection, the developed finisher has impact resistance.

### 4.3. Permeability Test

**Figure 9:**
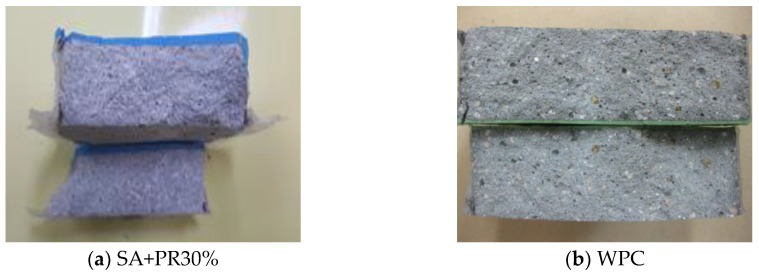
Test result of permeability.

The top coated cylinder specimen was under the constant water pressure head during the test according to KS F 4937. After test, the specimen was wiped out using filter paper and was split into two pieces to investigate water penetration through coated surface. Throughout the visual inspection between finisher and substrate of divided specimen profiles, as illustrated in Figure 9 below, it was determined that there no water penetration has occurred. Thus, it was found that the developed coating material has permeability against water.

### 4.4. Abrasive Wear Resistance to Wheel-Moving Test

**Figure 10:**
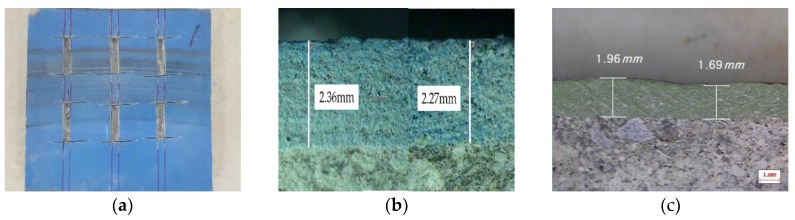
Test result of abrasive wear resistance. (**a**) Collection for depth measurement. (**b**) Surface thickness difference before and after the test in SA+PR30%. (**c**) Surface thickness difference before and after the test in WPC.

Abrasive wear resistance was validated by the cycling wheel test and the test was performed up to 80,000 cycles. During the wheel cycling, the visual observations were performed every 10,000 rotations in order to check for the appearance of pits, cracks, grounding, and spalling. The clearance at the surface of specimen was maintained up to 80,000 circuits. Additionally, an optical microscope was applied to calculate lost outward thickness resulted from tractional force of sand and tires, and the mean lost depth of all the collected specimens (Figure 10a) was below 0.1 mm from the original depth as shown in Figure 10b. The WPC lost 0.27 mm mean depth from the original depth. It showed 2.7 times higher resistance value compared to the recent developed finisher. From the test results it was found that the developed finisher complies with the acceptance of abrasive wear resistance to wheel moving test and is durable against the frictional loads from moving wheel.

### 4.5. Pollutant Emission Test

**Figure 11:**
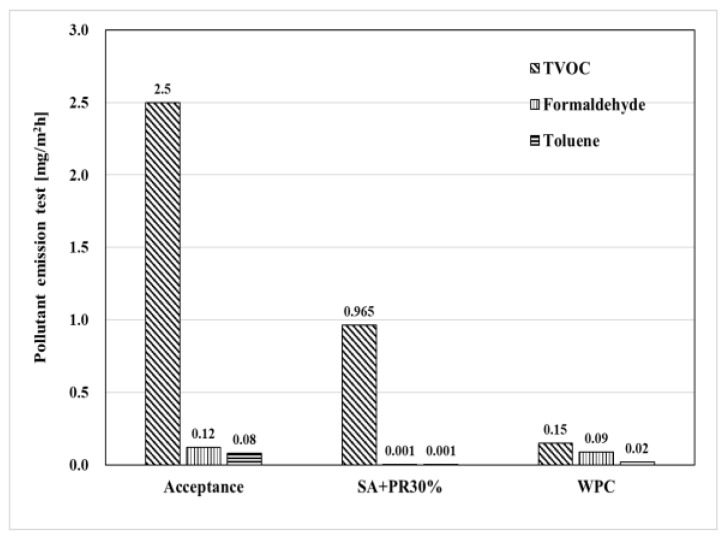
Pollutant emission result.

The samples of the developed finisher were placed into small chambers and the emitted amount of TVOC, formaldehyde, and toluene was determined according to KS I ISO 16000-3 and 16000-6. The emission test was fundamentally designed to determine compliance of the material to the ‘Indoor Air Quality Control Act’ in Korea. Figure 11 shows the test results of pollutant emission and the emitted quantity of all the test items were far below the requirements of each element.

## 5. Discussion

Throughout all the outcomes of evaluations, SA+PR30% and WPC satisfied both physical and pollutant emission tests following regulated Korea and British Standards as shown in Table 9. The results of adhesion strength was 1.05, 1.11, and 1.35 times higher than the epoxy, urethane, and WPC, respectively, and the wheel moving test showed 2.7 times higher values than the WPC. As described in the film formation mechanism of waterborne coatings by Swarts et al. [11], film formation occurred with a relatively slower water drying process compared to solvent-based coatings.

Amongst the abovementioned properties of the polyamide, good hydrophilicity could boost up the duration of film formation mechanism of the SA. Moreover, the rest of the properties of polyamide could promote the structural properties such as adhesion strength, abrasive wear, impact resistance, and permeability. It should be noted that polyamide has a cohesive effect to enhance mechanical properties when compounded with silicon acrylic, this is because the developed finisher is waterborne: it has more advantages for safety in terms of pollutant emission compared with conventional finishers such as epoxy and urethane finishers. It is also very important to consider what kind of finishers should be used for the car park slab in cases of fire. As results of the pollutant test, even though the TVOC emission from the SA+PR30% was a bit worse, the formaldehyde and toluene emission was better than the WPC. The reason that TVOC was a bit high could be attributed to impure SiO_2_. Despite all the benefits of PR, mix designs of finishers were planned to combine with up to 30% of PR because of cost-effectiveness. Since the adhesion strength increased proportionally with PR, it is expected that cracking at the surface of the finisher could develop during film formation. This is because an excessive amount of PR absorbs residual water in SA before proper formation of coating. Thus, it should be recommended to investigate the optimal amount of PR by further experiments in the future.

## 6. Conclusions

In order to develop a waterborne surface finisher with improved service life and low TVOC emission for car park slabs, the performance of the developed surface finisher was determined using the evaluation criteria from KS F 4937. The main results obtained from limited experiments are summarized as follows.

1. The finisher of this study consists of silicon acrylic, SiO_2_, water, and polyamide. After mixing these materials, performance evaluation such as the adhesion, impact resistance and other tests was carried out by criteria of KS F 4937. The mix ratio of the quality standard was silicon acrylic: SiO_2_:water:PR = 1:1:0.3:0.3.
2. In terms of adhesion strength, the waterborne silicon acrylic emulsion itself exceeded the bonding strength acceptance criterion (1.2 N/mm^2^) and the adhesion strength increased proportionally with polyamide. After the comparison with SA+PR, urethane, and epoxy finishers, SA+PR30% was determined as a potential replacement of the solvent-borne finishers. Compared to the WPC finisher, the developed finisher exhibited the adhesive strength of 1.34 times higher.
3. SA+PR30% satisfied the evaluation criteria of other tests in KS F 4937. In particular, the developed finisher had 2.7 times higher wheel moving resistance than the WPC finisher. Additionally, it was confirmed that the developed finisher could be an alternative finisher of urethane and epoxy finishers with less emission of TVOC.

It would be necessary to evaluate the cost effectiveness and perform more safety tests such as gas toxicity, heavy metal, chemical resistance, etc., through the future research for the car park slab finishers.

## Figures and Tables

**Table 1:** Comparison between Korean and European standards.

Evaluation Criteria		Korean	European
		Requirement	Requirement
Adhesion by pull-off test		≥1.2 N/mm^2^	≥1.5 N/mm^2^ (with or without trafficking) (BS EN 1542)
Impact resistance test		Pit, crack, grounding, and spalling should not occurr	Class II: ≥10 Nm (ISO 6272-1)
Permeability test		No water penetration through the surface	w < 0.1 kg/m^2^ × 0.5 h (BS EN 1062-3)
Abrasive wear resistance to wheel-moving test(300 kg, 80,000 cycles)	Status of surface	Pit, crack, grounding, and spalling should not occurr and the under layer should not be observed	weight loss < 3000 mg using H22 wheel, 1000 cycles, load 1000 g (ISO 5470-1)
	Lost surface thickness	≤3mm	-
Pollutant emission test	Total VOC	≤2.5 mg/m^2^·h	-
	Formaldehyde	≤0.5 mg/m^2^·h	-
	Toluene	≤0.08 mg/m^2^·h	-

**Table 2:** Chemical composition of silicon acrylic surface finisher.

Item	Chemical Composition (%)			
Silicon acrylic	Silicon acrylic resin	Water	Polyoxyethylene pentylphenol ether	etc.
	46–48	52–54	1–2	less than 1

**Table 3:** Chemical composition of urethane surface finisher.

Item	Chemical Composition (%)					
Primer	Polyurethane prepolymer	Xylene		Acetic acid 2-ethoxyethanol		Methyl ethyl ketone
	35–45	45–55		3–7		2–6
Urethane resin	Polyurethane prepolymer			Dimethyl benzene		
	90–97			3–10		
Urethane resin hardener	Polypropylene glycol		Calcium carbonate		Dibutyl phthalate	
	10–20		50–60		10–20	

**Table 4:** Chemical composition of epoxy surface finisher.

Item	Chemical Composition (%)					
Epoxy resin(Toplayer)	Calcium carbonate	4, 4′-Bisphenol polymet	Benzyl alcohol	4-Heptanone, 2, 6-dimethyl	Xylene	Dodecylphen-ol
	50–60	30–40	0.01–5	0.01–5	0.01–5	0.01–5
Epoxy resin (Underlyer)	Diglycidyl Ether of Bisphenol A	Xylene (Mixed)	Dimethyl carbonate	Ethyl Bensene	Acetone	2-Propanol
	30–40	30–40	10–20	5–10	5–10	0.1–5

**Table 5:** Chemical composition of polyamide resin.

Item	Chemical Composition (%)		
Polyamide resin	Polyamide resin	Water	Sales secret
	20–30	70–80	0.01–5

**Table 6:** Water-based polyurethane finisher with cementitious powder (WPC).

Item	Mix Ratio			
WPC	Water-based polyurethane	hardener	Water	Cementitious powder
	1	2	0.6	3.43

**Table 7:** Mix design of epoxy and urethane.

Specification		Mix Ratio		
		Resin	Hardener	Thinner (%)
Epoxy	Under layer	3.5	1	10%
	Top layer	8	1	10%
Urethane	Primer	1	-	5%
	Urethane	1	1	5%
	Top coating	1	4	5%

**Table 8:** Mix design of silicon acrylic with polyamide.

Specification	Mix Ratio			
	Waterborne Silicon Acrylic Emulsion	Water	SiO_2_	Polyamide Resin
SA ^1)^	1	0.4	1	-
SA+PR ^2)^ 1%				0.01
SA+PR 3%				0.03
SA+PR 5%				0.05
SA+PR 7%				0.07
SA+PR 9%				0.09
SA+PR 10%				0.10
SA+PR 20%				0.20
SA+PR 30%				0.30

^1)^ SA: Silicon Acrylic, ^2)^ PR: Polyamide Resin.

**Table 9:** Comparative values of test results.

Test List	Results of Test		Notes
	SA+PR30%	WPC	
Adhesion by pull out test	3.79 N/mm^2^	2.8 N/mm^2^	Age: 14 days
Impact resistance	Passed		
Water permeability	Passed		
Abrasive wear resistance to wheel-moving test	0.1 mm	0.27 mm	
Pollutant emission test	TVOC: 0.965 Formaldehyde: 0.001 Toluene: 0.001	TVOC: 0.15 Formaldehyde: 0.09 Toluene: 0.02	-
